# Non-Surgical Management of Bilateral Adrenal Abscess in Neonates: Report of Two Cases

**DOI:** 10.21699/jns.v6i2.493

**Published:** 2017-04-15

**Authors:** Ankur Mandelia, JS Kishore, Rajnikant Yadav, Richa Lal

**Affiliations:** 1 Department of Pediatric Surgery, Sanjay Gandhi Post Graduate Institute of Medical Sciences (SGPGIMS), Lucknow, India; 2 Department of Radiodiagnosis, Sanjay Gandhi Post Graduate Institute of Medical Sciences (SGPGIMS), Lucknow, India

**Keywords:** Adrenal abscess, Neonatal, Bilateral, Non-surgical management

## Abstract

Adrenal hemorrhage is not uncommon in neonates but the development of an adrenal abscess is extremely rare. Bilateral adrenal abscess is even rarer with less than ten cases documented in the medical literature. It may have a fatal outcome if inadequately treated. Here, we present two cases of neonates with history of obstructed labour and meconium aspiration who presented with bilateral adrenal abscesses. The abscesses were successfully treated using ultrasound-guided percutaneous drainage along with administration of appropriate intravenous antibiotics.

## Introduction

Adrenal hemorrhage is not uncommon in neonates but the development of an adrenal abscess is extremely rare. Bilateral adrenal abscesses is even rarer with less than ten cases documented in the medical literature [1-5]. Neonates usually present with non-specific signs and symptoms which might result in delay in diagnosis and extension of the suppurative process to adjacent organs. Prompt diagnosis requires a high index of suspicion. Surgical drainage was the mainstay of treatment of neonatal adrenal abscess in the past. However, ultrasound guided percutaneous drainage with intravenous antibiotics results in a successful outcome while avoiding the need for laparotomy [6-7].

## CASE SERIES

**Case 1:** A 20-day-old male baby, weighing 3.5 kg was admitted with history of intolerance of fee-s and increasing abdominal distension for the past 5 days. The child had been irritable and vomiting repeatedly for the past 3 days. The baby was delivered at term by normal vaginal delivery at a peripheral hospital. There was history of obstructed labour and liquor was meconium stained. Baby at birth was non vigorous, needed resuscitation and ventilatory support for 3 days. There was no history of fever or prolonged jaundice. Ultrasound scans in antenatal period were normal. Examination revealed an irritable neonate with pallor and moderate abdominal distension. Abdominal palpation revealed firm, tender masses in bilateral flanks. 

**Figure 1:**
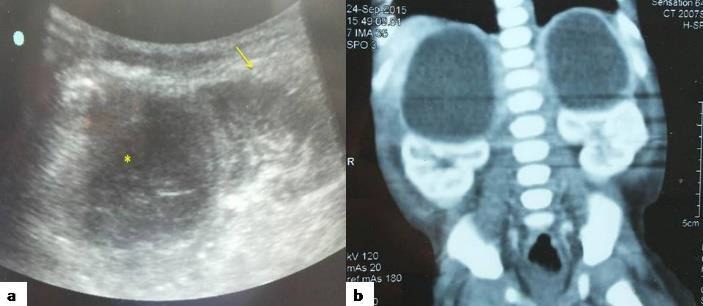
Case 1, a. Abdominal USG showing supra renal hypoechoic mass with internal echoes and debris (asterix) with inferior displacement of kidney (arrow) b. Abdominal computed tomography image (coronal section) showing well-circumscribed, thin-walled, peripheral-enhancing cystic lesions in bilateral suprarenal areas with inferior displacement of bilateral kidneys.

Initial laboratory data was as follows: haemoglobin 11.0 g/dl, WBC count 18,900/cu.mm with 65% neutrophils, 28% lymphocytes and 07% monocytes. Coagulation profile, renal function tests, liver function tests, 24 hour urinary vanillyl mandelic acid (VMA) and homovanillic acid (HVA) were normal. An abdominal ultrasonogram (US) showed bilateral supra renal hypo-echoic masses with internal echoes and debris, measuring 4.9 × 3.5 cm on the right side and 4.7 × 2.9 cm on the left side (Fig.1a). A contrast enhanced abdominal computed tomography (CECT) scan showed encysted collections with enhancing rims with fine internal septations in bilateral suprarenal areas with dimensions as seen on ultrasonogram (Fig. 1b). Both kidneys were displaced inferiorly by the lesions.

Based on the clinical presentation, laboratory data and imaging findings, a presumptive diagnosis of bilateral adrenal abscess was made and broad-spectrum empirical antibiotics (cefotaxime, amikacin) were started intra-venously. US-guided percutaneous aspiration was done through postero-lateral approach using a 20gauge needle. Thirty and 25 ml of thick, blood-stained, purulent fluid were aspirated from right and left suprarenal abscess respectively. The aspirate was sent for microscopy and bacterial culture. Microscopy demonstrated a predominance of polymorphonuclear leucocytes (95%) and gram-negative bacilli were noted on gram stain. Bacteriological cultures from the pus were positive for Klebsiella pneumoniae and antibiotic therapy was changed to meropenem and colistin as per culture sensitivity.

The clinical condition improved following US guided aspiration and the abdominal masses regressed on palpation. Laboratory tests showed normalising infection parameters. At 5 days after drainage, abdominal US showed a residual mass measuring 1.0×0.7 cm on the right side and 0.9 x 0.3 cm on the left side. The baby was discharged after 14 days of antibiotic therapy and further recovery was uneventful. At follow-up after 6 months, the child was doing well with no clinical signs or symptoms of adrenal insufficiency. Repeat serial US showed complete regression of the abscess cavities.

**Figure 2:**
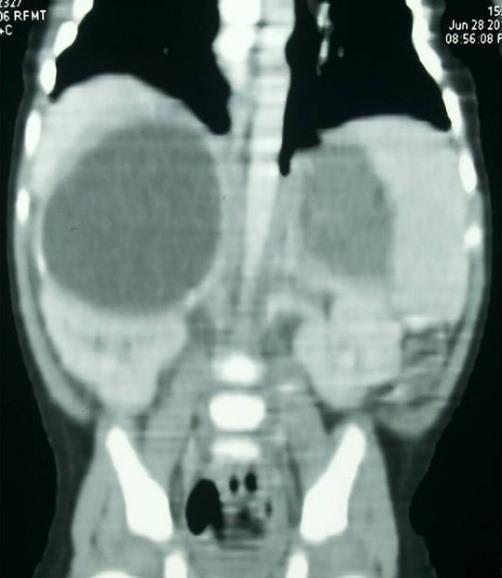
Case 2, Abdominal computed tomography image (coronal section) showing encysted collections with enhancing rims with fine internal septations in bilateral suprarenal areas, measuring 6.8 × 4.6 cm on the right side and 2.5 × 1.8 cm on the left side.

**Case 2: **A 28-day-old female baby, weighing 3.2 Kg was admitted with history of intermittent fever since birth. The baby was born at term by caesarean section at a private hospital. There was history of obstructed labour and liquor was meconium stained. The baby was admitted in the neonatal intensive care unit for observation and was discharged home after 72 hours. The parents complained of intermittent, low grade fever with irritability since discharge. There was no history of vomiting or persistent jaundice. Abdominal examination was unremarkable. Investigations done outside showed persistent leucocytosis (23,800/cu.mm with 65% neutrophils, 26% lymphocytes and 06% monocytes) and raised C-reactive protein (6.2 mg/dl). Coagulation profile, renal function tests, liver function tests, 24 hour urinary VMA and HVA were normal. CECT scan showed encysted collections with enhancing rims with fine internal septations in bilateral suprarenal areas measuring 6.8 × 4.6 cm on the right side and 2.5 × 1.8 cm on the left side (Fig. 2).

Broad-spectrum empirical antibiotics (Piperacillin-tazobactam, amikacin) were started intra-venously. US-guided percutaneous pig tail drain insertion was done in the larger cavity on the right side and single time needle aspiration was done on the left side. Sixty and 15 ml of thick, blood-stained, purulent fluid were aspirated from right and left suprarenal abscess respectively.

The fever subsided after drainage of pus and clinical condition improved. The right sided pig tail drain was removed after 72 hours. Bacteriological cultures from the pus were positive for Escherichia coli, sensitive to piperacillin-tazobactam and meropenem. The baby was discharged after 14 days of antibiotic therapy. At discharge, the leucocyte count and C Reactive Protein levels had normalised. Repeat US scan at one month showed complete regression of the abscess cavities. At follow-up after 3 months, the child was doing well with no clinical signs or symptoms of adrenal insufficiency.

## Discussion

Neonatal adrenal abscess is difficult to diagnose as the condition is rare and can present with non-specific signs and symptoms. The differential diagnosis of an adrenal collection includes hemorrhage, cyst, neuroblastoma, Wilms’ tumor, renal duplication with dilatation of the upper segment and hydronephrosis [7]. Prompt diagnosis and non-surgical treatment of an adrenal abscess may lead to disease resolution without the need for laparotomy [4-6].

Two theories have been proposed regarding the development of a neonatal adrenal abscesses: hematogenous bacterial seeding of a normal adrenal gland, and seeding of an adrenal hemorrhage with subsequent abscess formation [3.^6^.^7^]. However, the etiology may be unclear in some cases. It is likely that most adrenal abscesses result from adrenal hemorrhage associated with a traumatic or difficult delivery, hypoxia, sepsis, or coagulopathy [7]. In our patients, there was a history of prolonged labor, meconium aspiration with perinatal hypoxia. All these features suggest that the adrenal abscesses resulted from bacterial seeding of neonatal adrenal hemorrhage. Microbiological examination of abscess material revealed E. coli or S. aureus in most reported cases, but Streptococcus, Bacteriodes, Echovirus, and Herpes simplex virus have also been isolated [6,8,9]. Our cases were positive for Klebsiella pneumonia (Case 1) and E.coli (Case 2).

In the past, neonatal adrenal abscesses were treated with surgical exploration and drainage, which have been recommended as the therapies of choice for larger lesions or when diagnosis is unclear [3,8]. Due to technical developments, less invasive procedures may increasingly suffice. In both our cases, the abscess resolved with US-guided percutaneous drainage with appropriate antibiotic therapy, and no complications occurred during follow-up. The successful resolution of bilateral adrenal abscess with percutaneous drainage and intravenous antibiotics has also been reported by other authors [1,4,5]. 

To conclude, US-guided percutaneous drainage of adrenal abscess along with appropriate antibiotic therapy is safe and effective treatment which avoids the morbidity of laparotomy.

## Footnotes

**Source of Support:** None

**Conflict of Interest:** None
